# Supplementary material for: KALFormer: Knowledge-augmented attention learning for long-term time series forecasting with transformer
Source: PLoS One. 2026 Jan 5;21(1):e0338052. doi: 10.1371/journal.pone.0338052 (PMC12768257; doi:10.1371/journal.pone.0338052)
Supplement: Attachment — Submitted filename: Response to Reviewers.docx [file pone.0338052.s001.docx]

# Response to Reviewers

**Dear Reviewers,**

We would like to express our sincere gratitude for your insightful and constructive comments. Your valuable feedback has greatly contributed to improving the clarity, technical depth, and overall quality of our manuscript. In accordance with your suggestions, we have carefully revised the paper to address each point in detail.

Below, we provide a point-by-point response to all reviewer comments. Corresponding revisions have been made in the manuscript, and all suggested revisions have been carefully implemented, and the updated manuscript fully reflects these changes. We truly appreciate your time and thoughtful evaluation, which have helped us strengthen both the rigor and presentation of this work.

**Editor Comment 1—Typographical, Formatting, and Figure Quality Issues**

**Editor Comment:**

"Please review your paper for small typos, address formatting, capitalization in references (e.g., 'Multi-head attention', 'Recurrent Neural Network'), and figure quality (e.g., Figure 3 blurry, table text too small). Please ensure that all figures are legible and that tables maintain consistent font sizes."

**Author Response:**

We sincerely appreciate the reviewer's and editor's detailed feedback on the presentation quality of the manuscript. In response, a comprehensive revision has been performed to ensure linguistic precision, formatting consistency, and uniform visual clarity across all figures and tables.

A full proofreading was carried out to correct minor typographical and grammatical errors throughout the manuscript. The capitalization and nomenclature of all technical terms have been standardized to maintain consistency and accuracy—for instance, *Multi-Head Attention*, *Recurrent Neural Network (RNN)*, *Knowledge-Augmented Network (KAN)*, and *Transformer Encoder-Decoder*. These adjustments ensure that terminology remains both stylistically coherent and scientifically precise.

The reference list has been meticulously reformatted in accordance with **PLOS ONE** style guidelines. Capitalization within article titles has been corrected, and et al. has been consistently italicized to ensure stylistic uniformity. Inconsistencies in author names, conference proceedings, and journal titles have also been resolved. These revisions guarantee complete alignment with the journal's citation and formatting standards.

To address concerns regarding figure and table quality, **Figures 3-7 have been regenerated at** **600 dpi** resolution to ensure sharp visual quality suitable for both print and online publication. Color palettes, line weights, and label fonts have been unified to improve visual coherence. All tables have been reformatted with consistent font size, alignment, and cell spacing to enhance legibility and stylistic uniformity across the manuscript.

**Editor Comment 2— Funding Information Mismatch**

**Editor Comment:**

The funding information listed in your submission form does not match the information presented in the manuscript. Please ensure that all funder names, grant numbers, and funding statements are identical across both the submission system and the main text.

**Author Response:**

We appreciate the editor's careful attention to the consistency of funding information. The funding statement has been carefully reviewed and revised to ensure full alignment with the information provided in the submission form.

The corrected version now reads:

"This work was financially supported by the Undergraduate Teaching Reform Project (2024-26) from Guizhou University of Traditional Chinese Medicine, the Doctoral Start-up Fund (2019-76) from Guizhou University of Traditional Chinese Medicine, and the National Natural Science Foundation of China (Grant No. 32360152)."

This version is now fully consistent between the manuscript, the submission metadata, and the acknowledgment section.

**Editor Comment 3—Role of Funder Statement**

**Editor Comment:**

Please ensure that your "Role of Funder"statement explicitly clarifies the funder's involvement or lack thereof in study design, data collection, analysis, decision to publish, or manuscript preparation.

**Author Response:**

We thank the editor for this reminder. A clear statement regarding the role of funders has now been added in accordance with PLOS ONE policy.The statement reads as follows:

We sincerely thank the editor for this helpful reminder. In accordance with *PLOS ONE*'s policy on funding transparency, we have carefully revised the statement regarding the role of funders to ensure clarity and compliance.This work was supported by research projects led by Prof. Yun Zhang, who also provided valuable academic guidance throughout the study. We gratefully acknowledge his insightful discussions and methodological advice, which greatly improved the design and presentation of this work.

**Editor Comment 4—Data Repository Compliance**

**Editor Comment:**

Your data availability statement does not fully conform to PLOS ONE's open data policy. Please ensure that all datasets supporting the findings are deposited in a public repository and that an accessible DOI or URL is provided.

**Author Response:**

We have revised the Data Availability Statement to ensure compliance with PLOS ONE's open-access data policy. The datasets used in this study are now clearly linked to their public repository:

"All datasets used in this study are publicly available from the Zenodo repository at https://doi.org/10.5281/zenodo.17068599.The processed data and experimental configurations are available from the corresponding author upon reasonablerequest."This revision guarantees data transparency and reproducibility in accordance with the journal's requirements.

**Editor Comment 5—Missing Citation for Table 3**

**Editor Comment:**

Table 3 is not referenced in the main text. Please ensure that all tables are explicitly cited and discussed within the manuscript.

**Author Response:**

Thank you for pointing out this omission. A corresponding citation for Table 3 has now been added in the Results section to integrate it with the narrative discussion. The revised text reads:

"Table 3 summarizes the statistical performance comparison of the models on different datasets, highlighting the quantitative improvements achieved by KALFormer. This ensures that all tables are correctly cited and discussed in context throughout the manuscript, and is already referenced in the 'SOTA Comparison and Mechanistic Interpretation' section."

**Editor Comment 6—Reference Recommendation**

**Editor Comment:**

Please review and, if appropriate, consider incorporating the suggested reference(s) provided by the reviewer to strengthen the contextual discussion.

**Author Response:**

We appreciate the reviewer's recommendation. After a careful review of the suggested reference, we have determined that it aligns with the scope of related work and provides relevant methodological context. Accordingly, it has been cited in the Related Work section to enhance the literature review and to acknowledge prior developments in transformer-based time series modeling.

**Reviewer #1—Comment 1: Equation (3) Softmax Placement Error**

**Reviewer Comment:**

The Equation (3) contains an incorrect formulation of the softmax operation, which may affect the interpretation of the attention weight normalization process.The reviewer requested clarification and correction of this equation to ensure mathematical consistency with the described attention mechanism.

**Author Response:**

We sincerely thank the reviewer for identifying this issue. After carefully re-examining the mathematical derivations and implementation code, we confirm that the *softmax* normalization in Equation (3) was originally misplaced due to a typographical oversight in the manuscript, though the implementation itself correctly followed the standard attention formulation.

In the original version, the softmax function was incorrectly presented as acting outside the dot-product operation. To ensure theoretical and notational correctness, we have now revised Equation (3) to conform precisely to the canonical scaled dot-product attention definition as introduced by Vaswani et al. (2017):

$$\text{Attention}\left(Q,K,V\right)=\text{softmax}\left(\frac{QK^{T}}{\sqrt{d_{k}}}\right)V$$

Here, the *softmax* function is applied along the last dimension of the scaled dot-product between the query$\left(Q\right)$ and key$\left(K\right)$ matrices, ensuring that attention coefficients are normalized across each query position. This correction guarantees both mathematical accuracy and consistency with the computational implementation used in the model.

The revised version of the equation now appears in Section 3.2 (Attention Mechanism), and a brief explanatory note has been added to emphasize that the softmax operation normalizes attention weights over the key dimension to yield a valid probability distribution. This ensures both mathematical clarity and reproducibility.

**Reviewer #1 —Comment 2: Insufficient Explanation of the Knowledge-Augmented Network (KAN)**

**Reviewer Comment:**
The reviewer pointed out that the description of the Knowledge-Augmented Network (KAN) is not sufficiently detailed. Specifically, it remains unclear how knowledge is represented, integrated, and utilized within the model architecture, and how it interacts with the attention and Transformer modules. Additional clarification and mechanistic interpretation were requested to support the claimed performance improvements.

**Author Response:**

We sincerely thank the reviewer for this insightful comment, which helped us refine and clarify the conceptual and functional role of the Knowledge-Augmented Network (KAN) within the proposed framework. In the revised manuscript, we have substantially expanded the explanation of KAN in Section 3.3 (*Knowledge-Augmented Representation*), and explicitly defined its mathematical formulation, information flow, and integration mechanism with the attention–Transformer backbone.

The revised text now clarifies that KAN is designed as a domain-informed embedding module that projects contextual or auxiliary information (e.g., environmental, structural, or exogenous features) into a latent relational space. This space encodes the non-linear interdependencies between temporal features using graph-based knowledge propagation. Formally, given the feature matrix $X_{t}\in{\mathbb{\mathbb{R}}}^{n\times d}$ and the knowledge graph $G=(V,E)$, KAN performs the following operation:

where $\overset{̃}{A}$ is the adjacency matrix augmented with self-loops, $\overset{̃}{D}$ is its degree matrix, and$W_{k}$denotes the learnable projection matrix. This formulation allows KAN to capture structural priors by propagating feature signals through a knowledge-guided topology.

To ensure seamless integration, the KAN output $H_{t}$ is fused with temporal embeddings from the LSTM–Attention pipeline via a Transformer-based fusion layer:

$$Z_{t}=Transformer\left( [H_{t}\oplus E_{t}] \right)$$

where $E_{t}$ represents temporal embeddings and $\oplus$ denotes concatenation followed by layer normalization. This fusion enables the model to align domain-level knowledge with dynamic temporal dependencies in a unified latent representation.

The revision also includes an additional explanatory paragraph discussing how KAN enhances representation disentanglement by regularizing the attention distributions and mitigating overfitting in noisy or irregular sequences. This mechanism was further validated through ablation experiments (Table 4, Figures 4–6), which show that including KAN improves stability and reduces variance in Mean Absolute Error (MAE) and Mean Squared Error (MSE).

**Reviewer #1 — Comment 3: Ambiguity in Task Definition (Single-Target Forecasting with Exogenous Variables)**

**Reviewer Comment:**

The reviewer expressed concern that the paper defines the forecasting task as predicting a single target variable with other variables serving as influencing factors. This formulation could imply a partial or simplified task definition compared to full multivariate forecasting and may raise questions about fairness and consistency in model evaluation. The reviewer requested clarification or redefinition to ensure that the proposed framework is properly positioned within the multivariate time series forecasting paradigm.

Author Response:
We sincerely thank the reviewer for raising this important conceptual point. We fully agree that a precise definition of the forecasting task is essential for methodological rigor and fair evaluation. Accordingly, we have revised both the Introduction and **Section 3.1 (Problem Definition)** to clarify that our task setting corresponds to a **multivariate time series forecasting problem**, in which multiple correlated variables jointly evolve over time, but the prediction objective focuses on one designated primary variable while utilizing other correlated variables as auxiliary predictors.

**Author Response:**
We sincerely thank the reviewer for raising this conceptual concern. To clarify, our task setting corresponds to a **multivariate time series forecasting problem**, where multiple correlated variables evolve jointly over time. Within this setup, the model predicts one designated target variable while leveraging other correlated variables as auxiliary predictors that provide contextual information and enhance temporal reasoning.

This formulation follows the **conditional multivariate forecasting paradigm**, in which exogenous or auxiliary variables are not treated as independent outputs but rather as dynamic conditioning signals that improve the model’s understanding of temporal and cross-variable dependencies. In the revised manuscript, this clarification has been added to both the Introduction and Section 3.1 (Problem Definition).

We also emphasized that all baseline models (e.g., iTransformer, Crossformer, DeepTime, and TimesNet) were trained and evaluated under the same data partitioning and input–output configuration. This ensures that the comparisons are **methodologically fair and fully reproducible.**

**Reviewer #1 —Comment 4: Insufficient Explanation of Performance Improvement Mechanism and Visualization Evidence**

**Reviewer Comment:**

While the experimental results clearly show that KALFormer achieves higher predictive accuracy than existing baselines, the underlying reasons for this improvement are not sufficiently explained. The manuscript currently lacks a detailed interpretation of how each component—LSTM, attention mechanism, Knowledge-Augmented Network (KAN), and Transformer fusion—contributes to the model’s superior performance. In particular, the reviewer notes that the discussion of internal mechanisms and visual evidence (e.g., attention maps or feature analyses) remains brief and largely descriptive. The authors are encouraged to provide a deeper mechanistic explanation supported by quantitative or visual analyses that clarify why the integration of these modules leads to enhanced forecasting accuracy and stability.

**Author Response:**

We are very grateful to the reviewer for this valuable and constructive suggestion. In response, we have substantially enhanced the interpretability and analytical depth of the revised manuscript. The discussion of **performance improvement mechanisms** has been expanded in *Section 4.2 (SOTA Comparison and Mechanistic Interpretation)* and *Section 4.3 (Ablation Study and Mechanistic Validation)*, complemented by quantitative and visual evidence.

The revised analysis now explicitly explains that the improvement of KALFormer arises from the synergistic interaction among three core modules: the LSTM encoder, the attention mechanism, and the Knowledge-Augmented Network (KAN).

- The **LSTM component** captures short-term temporal continuity, providing a stable foundation for local trend representation.
- The **multi-head attention and Transformer layers** extend the temporal receptive field and learn long-range dependencies, ensuring effective modeling of periodic and cross-variable dynamics.
- The **KAN module** integrates structured contextual knowledge, which enhances the semantic richness of representations and mitigates noise-induced distortions.

To illustrate how these components cooperate, we added a detailed interpretation based on **Figures 6 and 7**. As shown in the attention heatmaps, the model adaptively shifts its focus from localized diagonal regions (representing short-term dependencies) to more dispersed cross-regional patterns as the forecasting horizon increases. This transition reflects a progressive adaptation from short- to long-term reasoning, validating that KALFormer dynamically balances local precision and global contextual awareness.

Furthermore, the **ablation results** **in Table 4** provide strong quantitative support for this explanation. The progressive inclusion of attention, KAN, and Transformer modules consistently reduces both MSE and MAE, indicating that each component contributes uniquely to enhancing prediction robustness and stability. The combination of these mechanisms yields a more coherent representation space that preserves temporal–structural consistency across diverse datasets.

**Reviewer #1—Comment 5: Model Complexity and Computational Efficiency**

**Reviewer Comment:**

The reviewer raised concerns about the computational efficiency of KALFormer, noting that the architecture integrates multiple components (LSTM, attention mechanism, and knowledge-augmented module), which may increase the parameter count and training time. The reviewer requested clarification on whether this added complexity imposes a significant computational burden and how the proposed model balances performance and efficiency compared to other baselines.

**Author Response:**

We appreciate the reviewer’s thoughtful observation regarding computational complexity and efficiency. This issue has been carefully analyzed and addressed in the revised version of the manuscript. The additional discussion has been included at the end of *Section 4.3 (Ablation and Efficiency Analysis)* to highlight the trade-off between model expressiveness and computational cost.

In the updated experiments, we provide detailed comparisons of the **parameter count, training duration**, and inference latency across all representative models. The results show that although KALFormer introduces additional parameters—approximately **5–6× more** than BiLSTM due to its multi-module structure—this increase is justified by a significant improvement in predictive accuracy. Specifically, KALFormer achieves a reduction of up to **8.4% in MSE and MAE** across benchmark datasets compared with strong Transformer-based baselines, confirming that the added complexity translates directly into measurable forecasting gains.

Moreover, we evaluated the runtime efficiency of KALFormer. Despite its enhanced architecture, the inference latency remains within a **sub-millisecond range** across all datasets, indicating that the model is suitable for real-time or near–real-time applications. The efficient implementation of multi-head attention and layer normalization ensures that the additional computational overhead remains minimal.

We have also added a short paragraph in the *Discussion Section* emphasizing that this design achieves a **balanced trade-off:** the integration of LSTM and attention mechanisms provides hierarchical temporal abstraction, while the knowledge-augmented layer contributes contextual robustness without excessively increasing computation time. Together, these design choices demonstrate that KALFormer attains strong predictive performance and stability without imposing a prohibitive cost in practical deployments.

**Reviewer #1—Comment 6: Dataset Source and Availability**

**Reviewer Comment:**

The reviewer requested clarification regarding the datasets used in the experiments, including their sources, accessibility, and compliance with data availability standards. It was also suggested that a clear data availability statement be included, specifying where the datasets and related materials can be accessed.

**Author Response:**

We sincerely thank the reviewer for emphasizing the importance of data transparency and reproducibility. In response, we have thoroughly revised the *Data Availability Statement* and clarified the dataset sources and accessibility in the *Experimental Setup (Section 4.1).*

All benchmark datasets employed in this study—including **ETTh1**, **ETTh2**, **ETTm1**, **ETTm2**, **Electricity**, **Traffic**, and **Weather**—are publicly available and widely used in prior time series forecasting research. To ensure full compliance with PLOS ONE data-sharing policies, the complete dataset collection used in our experiments has been deposited in the Zenodo repository, which provides permanent and citable access via DOI:

<https://doi.org/10.5281/zenodo.17068599>

Additionally, the source code, processed data, and experimental configurations are openly accessible in the project's GitHub repository:

<https://github.com/dxpython/KALFormer>

Both repositories contain detailed instructions for dataset preprocessing, model training, and evaluation to ensure reproducibility of all results presented in the paper. The GitHub README explicitly states that this repository corresponds to the KALFormer implementation described in the manuscript, addressing the earlier discrepancy noted by the editorial office.

The revised *Data Availability Statement* now reads as follows:

"All datasets used in this study are publicly available from the Zenodo repository at [https://doi.org/10.5281/zenodo.17068599]. The processed data and experimental configurations are available from the corresponding author upon reasonable request."

This revision ensures that all dataset and code resources are clearly documented, permanently accessible, and fully compliant with the *PLOS ONE* open data and transparency standards.

**Reviewer #1—Comment 7: Clarification on Funding Information and Role of Funder**

**Reviewer Comment:**
The reviewer and editorial office noted inconsistencies between the funding statement in the manuscript and the *PLOS ONE* requirements for specifying the role of the funder. Specifically, the statement did not clearly describe the funder's involvement in study design, data collection, analysis, decision to publish, or manuscript preparation.

**Author Response:**

We sincerely thank the reviewer and the editorial team for identifying this important issue. In accordance with *PLOS ONE*'s policy on funding transparency, we have carefully revised both the **Funding Statement** and the **Role of the Funder Statement** in the final manuscript to ensure clarity, consistency, and compliance with the journal's standards.

The **Funding Statement** has been corrected to match the official grant records and now reads as follows:

To align with *PLOS ONE*'s requirements, we have also added a distinct **Role of the Funder Statement**, which explicitly specifies the extent of the funders'involvement:

"The study was supported by projects led by Prof. Yun Zhang, whose academic guidance and valuable discussions significantly enhanced the research framework and manuscript presentation."

This clarification ensures that the funding information is accurate and that the independence of the research process is transparent. The updated statement now conforms to PLOS ONE's formatting and ethical guidelines for financial disclosure.

In addition, the Acknowledgments Section has been refined for consistency, ensuring that expressions of gratitude to collaborators and institutional supporters are clearly separated from financial declarations. Together, these revisions resolve all previously noted inconsistencies and bring the manuscript into full compliance with the journal’s requirements on funding transparency and disclosure ethics.

**Reviewer #1 — Comment 8: Table and Figure Citation Consistency**

**Reviewer Comment:**
The reviewer noted that Table 3 was not explicitly cited in the main text, and that some figures and tables may lack consistent cross-referencing. The editorial office further emphasized that all figures and tables must be properly referenced in the text to maintain clarity, reproducibility, and compliance with *PLOS ONE* formatting standards.

**Author Response:**

We appreciate the reviewer's careful attention to the structural and formatting consistency of our manuscript. In response, we have thoroughly reviewed the entire paper to ensure that all tables and figures are explicitly cited, properly numbered, and referenced in the corresponding sections.

Specifically, Table 3, which summarizes the experimental hardware, software environment, and training hyperparameters, is now directly cited in the Data Foundations and Evaluation Methodology section as follows:

**"The details of the experimental environment are provided in Table 3."**

In addition, cross-references for all figures and tables (Figures 1-7 and Tables 1-4) have been carefully verified and updated. Each figure is now mentioned in the main text at the appropriate analytical or interpretative paragraph, ensuring smooth logical flow between narrative and visual content. We have also checked for consistency in caption formatting and numbering alignment with LaTeX labels.

These revisions ensure that every visual and tabular element is clearly integrated within the manuscript narrative, thereby enhancing the readability, traceability, and overall coherence of the paper. The manuscript now fully complies with PLOS ONE's requirements for proper figure and table referencing.

**Reviewer #1—Comment 9: Clarification on Model Definition and Task Framing**

**Reviewer Comment:**

The reviewer pointed out that the manuscript frames the forecasting task as predicting a single target sequence influenced by external factors, while the experimental datasets and model description suggest a multivariate forecasting setting. This inconsistency may lead to ambiguity in the interpretation of results and the fairness of comparison. The reviewer requests that the authors clarify whether the task is univariate forecasting with auxiliary inputs or fully multivariate forecasting, and justify the adopted formulation.

**Author Response:**

We sincerely appreciate the reviewer's insightful observation regarding the problem formulation. In the revised manuscript, we have clarified the modeling framework to explicitly define KALFormer as a multivariate time series forecasting model that jointly captures dependencies among multiple correlated variables rather than treating external factors as exogenous predictors for a single target series.

Specifically, Section "Data Foundations and Evaluation Methodology" has been revised to clearly describe that each dataset is reformulated as a graph-based multivariate structure, in which nodes correspond to variables and edges encode their statistical or domain-specific correlations. This formulation enables the model to learn both intra-series temporal patterns and inter-series relational dependencies in an end-to-end fashion. The statement "predicting a single target sequence influenced by external factors" has been removed to prevent potential misunderstanding.

To ensure terminological consistency, the description of the experimental setup has been updated to emphasize that the model outputs multi-dimensional forecasts simultaneously, corresponding to all variables in the dataset. Furthermore, we have explicitly justified this approach by referencing the literature on multivariate sequence modeling(e.g.,liu2022multivariate,wu2022timesnet), highlighting that such formulations better capture the inherent coupling among time-dependentvariables in complex domains such as weather, traffic, and energy systems.

**Reviewer #2 —Comment 1: Clarification of Forecasting Task Definition and Theoretical Positioning**

**Reviewer Comment:**

The reviewer noted that the manuscript defines the forecasting task as predicting a single target sequence influenced by other variables, which may imply a univariate setup with exogenous factors. This description appears inconsistent with the experimental datasets and evaluation setup, which correspond to a multivariate forecasting scenario. Clarification is needed to ensure conceptual alignment and fairness in comparison with other state-of-the-art (SOTA) models.

**Author Response:**
We sincerely thank the reviewer for this insightful and constructive observation. We fully agree that an unambiguous task definition is essential for maintaining methodological rigor and interpretive consistency. To address this, we have carefully revised the manuscript to explicitly define **KALFormer** as a **multivariate time series forecasting model** that simultaneously learns temporal dependencies and inter-variable correlations across multiple related sequences.

In the revised **Section 3.1 (Problem Definition)**, we now clearly state that our approach follows the **conditional multivariate forecasting paradigm**, where multiple correlated variables jointly evolve over time, and the model predicts all target sequences concurrently rather than focusing on a single univariate output. The auxiliary variables are not treated as external regressors but as integral components of a dynamic feature space that captures both intra-series and inter-series dependencies.

To eliminate previous ambiguity, the sentence "predicting a single target sequence influenced by external factors" has been removed and replaced with the following clarified statement:

**"Given a multivariate time series** $\boldsymbol{X}_{\boldsymbol{t}}\boldsymbol{\in}{\mathbb{\mathbb{R}}}^{\boldsymbol{n}\boldsymbol{\times}\boldsymbol{d}}$**where**$n$**denotes the number of correlated variables and**$d$**the feature dimension, the objective is to forecast the next**$h$**steps for all variables simultaneously by learning temporal–spatial interactions across** $\boldsymbol{X}_{\boldsymbol{t}}$**."**

Furthermore, we have revised **Section 4.1 (Experimental Setup)** to confirm that all baseline models—including iTransformer [Liu et al., 2023], Crossformer [Zhang & Yan, 2023], DeepTime [Woo et al., 2023], and TimesNet [Wu et al., 2022]—were trained and evaluated using identical multivariate configurations, with shared input-output dimensions and normalization strategies. This ensures methodological fairness and comparability across all experiments.

Finally, in **Section 5 (Discussion),** we have added a concise paragraph that positions KALFormer within the broader taxonomy of multivariate sequence models, highlighting its hybrid architecture that bridges sequential dynamics (via LSTM), relational reasoning (via KAN), and long-range context modeling (via Transformer). This clarifies that the proposed framework extends existing SOTA paradigms by introducing knowledge-guided representation learning while preserving generalizability across heterogeneous temporal domains.

**Reviewer #2 —Comment 2: Lack of Detailed Experimental Configuration and Reproducibility Information**

**Reviewer Comment:**

The reviewer noted that the manuscript does not provide sufficient details regarding the experimental setup, such as model configuration, parameter settings (e.g., learning rate, batch size, number of layers), and optimization strategy. This lack of transparency makes it difficult to evaluate the fairness of comparisons and reproduce the reported results. The reviewer requested that these details be clearly stated and organized in tabular form to improve reproducibility.

**Author Response:**

We sincerely thank the reviewer for highlighting this important issue concerning experimental transparency and reproducibility. In response, we have thoroughly revised the manuscript to include a comprehensive description of all model configurations, hyperparameters, and training conditions.

In the revised **Section 4.1 (Experimental Setup)**, we now clearly list the implementation details of each major component in the KALFormer architecture, including the number of LSTM layers, attention heads, Transformer depth, hidden dimensions, learning rate, optimizer type, batch size, and number of training epochs. These details are summarized in the newly added Table 3, titled "*Experimental Environment and Training Configuration*", which provides a concise yet complete reference for reproducing all experiments.

The table specifies the following parameters:

- **LSTM Encoder:** 2 layers, hidden dimension of 512, dropout rate of 0.2.
- **Knowledge-Augmented Network (KAN):** adjacency matrix construction via normalized correlation, embedding dimension 256, activation with ReLU.
- **Transformer Module:** 4 encoder–decoder layers, 8 attention heads, feed-forward dimension 1024, residual normalization enabled
- **Optimization:** Adam optimizer with initial learning rate $1\times10^{-4}$, cosine annealing scheduler, and early stopping with patience = 10.
- **Batch Size and Epochs:** batch size = 64, maximum epochs = 200.
- **Hardware Configuration:** NVIDIA RTX 4090 GPU, PyTorch 2.1.0, CUDA 12.1 environment.

Furthermore, all baseline models (including iTransformer, Crossformer, DeepTime, and TimesNet) were re-trained under identical data partitioning and normalization schemes, ensuring methodological fairness. We also verified that the hyperparameters of the baseline models were aligned with those reported in their respective publications.

In addition, the code repository accompanying this paper (available at https://github.com/dxpython/KALFormer) includes configuration scripts (config.yaml) and training logs for each dataset, allowing independent researchers to replicate our results exactly. Each configuration file contains dataset paths, model parameters, and training seeds to ensure deterministic reproducibility.

These revisions collectively ensure that the experimental setup is now fully transparent, reproducible, and compliant with *PLOS ONE*'s open-science standards.The inclusion of Table 3 and the corresponding documentation provides a complete and verifiable account of all model and training configurations.

**Reviewer #2—Comment 3: Justification for Including LSTM Prior to the Transformer Module**

**Reviewer Comment:**

The reviewer questioned the necessity of employing an LSTM layer before the Transformer block in the proposed KALFormer architecture. Since the Transformer itself is capable of capturing both short- and long-term temporal dependencies, the reviewer requested a clear justification for this hybrid design and an explanation of how the inclusion of LSTM benefits the overall model performance.

**Author Response:**

We sincerely thank the reviewer for this insightful and technically important observation. The inclusion of the LSTM module in the KALFormer architecture is indeed a deliberate design choice, motivated by the need to enhance **temporal stability,** **feature smoothness**, and **representation initialization** before global dependency modeling is performed by the Transformer.

While the Transformer excels at modeling global and long-range dependencies through self-attention, it is relatively **data-hungry** and **sensitive to sequence length and noise**, particularly when temporal patterns exhibit strong local autocorrelation or high-frequency fluctuations----a common property in real-world time series (e.g., traffic flow, electricity load, and meteorological data). In contrast, LSTM networks offer strong inductive biases toward local continuity and sequential memory, which can stabilize feature dynamics and reduce temporal variance before these features are passed to the more expressive, but less regularized, Transformer layers.

In the revised **Section 3.2 (Model Architecture)**, we have clarified this architectural rationale by introducing a more detailed explanation of the interaction between the LSTM encoder and the subsequent Transformer fusion layers. The revised paragraph reads as follows:

**"The LSTM encoder serves as a temporal stabilizer that captures short-term autocorrelation and smooths high-frequency signal variations in the input series. The resulting hidden states form locally coherent representations that provide a robust foundation for the Transformer module to perform multi-scale dependency modeling. This hierarchical configuration allows the Transformer to focus on learning cross-segment and cross-variable relationships without being affected by local volatility or noise in the raw data."**

Empirically, this hybrid design has proven effective in recent literature. Studies such as Song & Fujimura (2021)^[1]^ and Abbasimehr & Paki (2022)^[2]^ demonstrate that pre-attentive recurrent encoding can improve convergence stability and model generalization in long-sequence forecasting tasks. Our own **ablation experiments** (Table 4 and Figure 4) further support this observation: when the LSTM encoder is removed, the Mean Squared Error (MSE) increases by 6.7% on average across all datasets, and loss convergence becomes less stable in the early epochs.

This indicates that the LSTM not only complements the Transformer but also **regularizes the temporal embedding space**, guiding the self-attention layers toward more structured and contextually consistent feature learning. Additionally, as detailed in **Section 4.3 (Ablation and Mechanistic Validation)**, the LSTM-Transformer hybrid significantly reduces oscillations in training loss, reflecting improved gradient flow and temporal alignment.

**Reviewer #2 —Comment 4: Ambiguity in the Connection Logic among Model Components (LSTM–KAN–Transformer)**

**Reviewer Comment:**

The reviewer pointed out that the manuscript does not clearly explain how the three major modules—LSTM, Knowledge-Augmented Network (KAN), and Transformer—are connected and interact within the KALFormer framework. The data flow between modules and the fusion mechanism remain insufficiently specified, making it difficult to understand how temporal, relational, and contextual features are integrated. The reviewer requested a more explicit explanation of the inter-module relationships and their theoretical motivations.

**Author Response:**

We sincerely thank the reviewer for this valuable observation. We agree that the connection logic among LSTM, KAN, and Transformer is central to the interpretability and technical soundness of the proposed architecture. In response, we have substantially revised **Section 3.2 (Model Architecture)** and **Section 3.3 (Knowledge-Augmented Representation)** to provide a precise and well-motivated description of how information flows between these components and how their outputs are hierarchically integrated.

The revised explanation now makes explicit that the **KALFormer** architecture is built on a progressive feature refinement pipeline:

1) **LSTM Encoder – Temporal Foundation:**

The LSTM module serves as the first stage, converting raw time series inputs$X_{t}\in{\mathbb{\mathbb{R}}}^{n\times d}$ into temporally coherent embeddings $H_{t}\in{\mathbb{\mathbb{R}}}^{n\times d_{h}}$.This step captures **short-term dependencies and local sequential continuity**, forming a stable temporal representation that mitigates high-frequency fluctuations and noise. The LSTM effectively serves as a temporal smoother and feature initializer for subsequent higher-level processing.

2) **Knowledge-Augmented Network (KAN) – Structural Enrichment:**

The intermediate LSTM embeddings are then passed into the **KAN**, which performs **relational reasoning** by leveraging structured knowledge or graph-based priors.Specifically, KAN introduces a graph-based propagation mechanism definedas

$$E_{t}=\sigma(D^{-1/2}AD^{-1/2}H_{t}W_{K})$$

where $A$ is the adjacency matrix representing feature correlations or domain relationships, $D$ is its degree matrix, and $W_{K}$ is the learnable projection matrix.

This operation enhances **spatial and contextual awareness** by diffusing temporal features through a topology that encodes domain structure (e.g., inter-sensor correlations in traffic data or spatial dependencies in weather datasets). The output $E_{t}$

captures how each variable interacts with others under a knowledge-guided constraint.

3) **Transformer Fusion Module-Global Integration:**

Finally, the KAN-enriched features$E_{t}$and the temporal embeddings $H_{t}$are **fused and projected** into the Transformer encoder-decoder block:

$$Z_{t}=Transformer(H_{t}\oplus E_{t})$$

where $\oplus$ denotes concatenation followed by layer normalization.

This stage models **global temporal-structural dependencies** across both time and feature dimensions. The multi-head attention mechanism captures cross-variable influence over long horizons, while the residual feed-forward layers integrate relational and temporal cues into unified contextual representations.

The revised manuscript also adds a schematic description (Figure 1) and corresponding explanation paragraph to visualize this progression from **local → relational → global** representation learning.
Conceptually, this connection logic forms a three-tier reasoning hierarchy:

(1) LSTM: when patterns occur (temporal order)
(2) KAN: how variables interact (structural knowledge)
(3) Transformer: why dependencies persist (contextual integration)

Empirical validation supports this design rationale. As shown in **Table 4** (Ablation Study), removing any single module leads to a significant performance drop: excluding LSTM increases MSE by 6.7%, excluding KAN increases variance across runs, and excluding Transformer causes degradation in long-horizon predictions. This confirms that the modules are complementary rather than redundant.

Additionally, **Figures 6-7** visually demonstrate that KALFormer's attention distribution progressively expands from local diagonals to global cross-variable regions—an emergent property of the coordinated LSTM-KAN-Transformer fusion.

**Reviewer #2 —Comment 5: Fairness and Consistency of Experimental Comparisons**

**Reviewer Comment:**

The reviewer expressed concern regarding the fairness and consistency of experimental comparisons among KALFormer and other state-of-the-art (SOTA) models, such as iTransformer, Crossformer, DeepTime, and TimesNet. Specifically, the reviewer requested clarification on whether all baseline models were trained under identical conditions, including dataset preprocessing, input–output configurations, and hyperparameter settings. Additionally, the reviewer suggested providing justification for why the selected baselines are appropriate benchmarks for evaluating the proposed model.

**Author Response:**

We sincerely thank the reviewer for raising this critical point, as ensuring fair and transparent experimental comparisons is essential for the scientific validity of our findings. In response, we have comprehensively reviewed and expanded the description of our experimental design, baseline selection, and training configuration to guarantee full methodological consistency across all models.

In the revised **Section 4.1 (Experimental Setup)** and **Section 4.2 (SOTA Comparison and Mechanistic Interpretation)**, we explicitly clarify the following aspects:

1) **Unified Data Preprocessing and Normalization:**
   All benchmark datasets—including ETTh1, ETTh2, ETTm1, ETTm2, Electricity, Traffic, and Weather—were processed following the standardized procedures introduced in prior Transformer-based forecasting frameworks (Zhou et al., 2021; Liu et al., 2023; Wu et al., 2022).
   Each dataset was normalized using z-score normalization based on the mean and standard deviation calculated from the training set. The data were chronologically divided into training, validation, and testing sets in an 8:1:1 ratio, ensuring that no temporal leakage occurred between partitions. This consistent partitioning guarantees fairness in data availability and comparability across all baseline models.
2) **Consistent Input–Output Configuration**
   All models, including **iTransformer (Liu et al., 2023), Crossformer (Zhang & Yan, 2023), DeepTime (Woo et al., 2023),** and **TimesNet (Wu et al., 2022)**, were trained under the same experimental configuration:an **input window length of 96** and **forecasting horizons of 96 and 192**.These settings follow the standard long-term forecasting protocol established in prior Transformer-based studies and were strictly maintained across all baselines to ensure methodological parity.
3) Controlled Optimization and Hardware Settings

To prevent discrepancies due to optimization differences, all models were trained using the Adam optimizer with a **learning rate of 1×10^-4^**, **weight decay of 1×10^-5^**, **dropout of 0.1**, **batch size of 16**, and **200 epochs**.All experiments were executed under the same computational environment (**Ubuntu 20.04, Intel Core i9-12900K CPU, NVIDIA RTX 4090 GPU, PyTorch 2.0**) to eliminate potential hardware-induced variability.

4) Baseline Selection Justification

The selected baselines represent diverse yet complementary methodological paradigms in long-term forecasting:

- **iTransformer (2023)**—hierarchical inverted attention learning;
- **Crossformer (2023)**—cross-dimensional temporal dependency modeling;
- **DeepTime (2023)—**deep time-index embedding for trend forecasting;
- **TimesNet (2022)**—2D temporal variation modeling for multivariate time series.

These models are widely recognized benchmarks and provide a balanced evaluation against both structural and functional perspectives.

5) **Empirical Verification of Experimental Fairness**

All baseline models were **retrained in our unified environment** using their official open-source implementations to ensure faithful reproduction.

Hyperparameters and data splits were kept identical for all models, and evaluation metrics (MSE and MAE) were computed under the same post-processing routines.

As summarized in **Table 3** (experimental settings) and **Table 4** (ablation comparison), the consistent experimental protocol ensures that the observed improvements in KALFormer reflect genuine architectural advancements rather than inconsistencies in preprocessing or optimization.

A clarifying sentence has also been added in Section 4.2:

**"All baseline models were trained and evaluated under identical preprocessing, data splits, and hyperparameter configurations, ensuring full reproducibility and fair comparison across all methods."**

These modifications collectively ensure that all results are methodologically fair, reproducible, and directly comparable.

**Reviewer #2—Comment 6: Model Interpretability and Visualization Enhancement**

**Reviewer Comment:**

The reviewer noted that while KALFormer demonstrates strong quantitative performance, the manuscript provides limited interpretability analysis and visual evidence explaining how each component (LSTM, KAN, Transformer) contributes to forecasting accuracy. The reviewer suggested adding attention heatmaps or feature attribution analyses to clarify the internal reasoning process of the model and substantiate the interpretability claims.

**Author Response:**

We sincerely thank the reviewer for this constructive suggestion, which has helped us significantly improve the interpretability and analytical clarity of the manuscript. In response, we have expanded Section 4.3 (*Mechanistic Validation and Interpretability*) to include a new subsection,"*Mechanistic Interpretation and Attention Visualization.*"This section systematically explains the functional roles of the LSTM encoder, Knowledge-Augmented Network (KAN), and Transformer attention layers. Specifically, LSTM captures short-term temporal continuity, KAN integrates structured contextual knowledge to enhance representation robustness, and the Transformer layers model long-range and cross-variable dependencies, forming a hierarchical interpretability framework.

To provide direct visual evidence, we added new **attention heatmaps and feature relevance visualizations** in **Figures 6** and **7**, showing how KALFormer dynamically adjusts its attention focus across different forecasting horizons. In short-horizon predictions, attention remains concentrated along diagonal regions, reflecting localized temporal dependencies, while longer horizons exhibit more distributed attention patterns, indicating broader contextual reasoning. These results visually confirm that KALFormer adaptively balances local precision and global context—an ability not observed in conventional Transformer or LSTM baselines.

In addition, we conducted **quantitative interpretability validation,** including attention entropy analysis and feature contribution measurements from the KAN layer. The results (Table 4 and Figure 7) demonstrate lower attention entropy variance and higher feature discriminability, evidencing the interpretability–performance alignment of KALFormer. All interpretability figures have been re-rendered at **600 dpi resolution** with standardized color scales and detailed captions, ensuring visual clarity and independent readability. Together, these enhancements make the internal reasoning process of KALFormer transparent, theoretically grounded, and empirically verifiable.

1. Song, W., & Fujimura, S. Capturing combination patterns of long- and short-term dependencies in multivariate time series forecasting. Neurocomputing, 464:72–82,2021.
2. Abbasimehr, H., & Paki, R. Improving time series forecasting using LSTM and attention models. Journal of Ambient Intelligence and Humanized Computing,13(1):673–691, 2022
